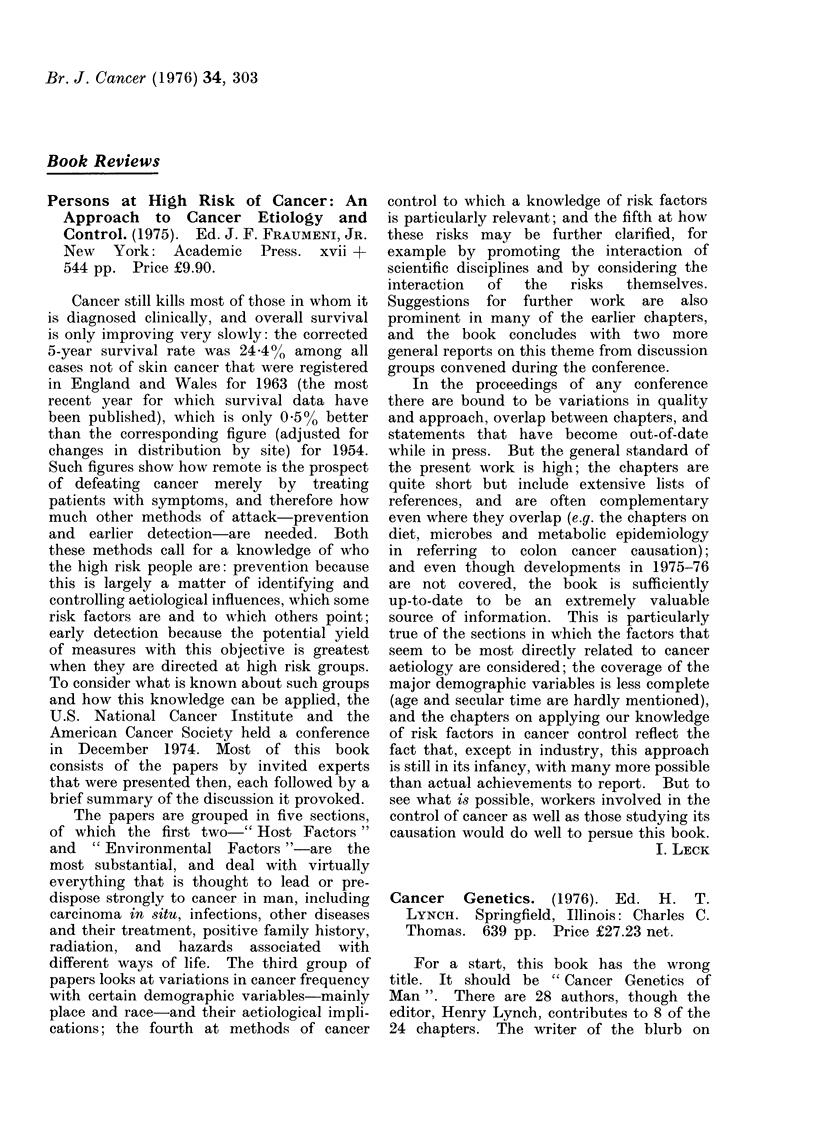# Persons at High Risk of Cancer: An Approach to Cancer Etiology and Control

**Published:** 1976-09

**Authors:** I. Leck


*Br. J. Cancer* (1976) **34**, 303

### *Book Reviews*

**Persons at High Risk of Cancer: An Approach to Cancer Etiology and Control.** (1975). Ed. J. F. Fraumeni, Jr. New York: Academic Press. xvii + 544 pp. Price £9.90.

Cancer still kills most of those in whom it is diagnosed clinically, and overall survival is only improving very slowly: the corrected 5-year survival rate was 24·4% among all cases not of skin cancer that were registered in England and Wales for 1963 (the most recent year for which survival data have been published), which is only 0·5% better than the corresponding figure (adjusted for changes in distribution by site) for 1954. Such figures show how remote is the prospect of defeating cancer merely by treating patients with symptoms, and therefore how much other methods of attack—prevention and earlier detection—are needed. Both these methods call for a knowledge of who the high risk people are: prevention because this is largely a matter of identifying and controlling aetiological influences, which some risk factors are and to which others point; early detection because the potential yield of measures with this objective is greatest when they are directed at high risk groups. To consider what is known about such groups and how this knowledge can be applied, the U.S. National Cancer Institute and the American Cancer Society held a conference in December 1974. Most of this book consists of the papers by invited experts that were presented then, each followed by a brief summary of the discussion it provoked.

The papers are grouped in five sections, of which the first two—" Host Factors " and " Environmental Factors "—are the most substantial, and deal with virtually everything that is thought to lead or predispose strongly to cancer in man, including carcinoma *in situ*, infections, other diseases and their treatment, positive family history, radiation, and hazards associated with different ways of life. The third group of papers looks at variations in cancer frequency with certain demographic variables—mainly place and race—and their aetiological implications; the fourth at methods of cancer control to which a knowledge of risk factors is particularly relevant; and the fifth at how these risks may be further clarified, for example by promoting the interaction of scientific disciplines and by considering the interaction of the risks themselves. Suggestions for further work are also prominent in many of the earlier chapters, and the book concludes with two more general reports on this theme from discussion groups convened during the conference.

In the proceedings of any conference there are bound to be variations in quality and approach, overlap between chapters, and statements that have become out-of-date while in press. But the general standard of the present work is high; the chapters are quite short but include extensive lists of references, and are often complementary even where they overlap (*e.g.* the chapters on diet, microbes and metabolic epidemiology in referring to colon cancer causation); and even though developments in 1975–76 are not covered, the book is sufficiently up-to-date to be an extremely valuable source of information. This is particularly true of the sections in which the factors that seem to be most directly related to cancer aetiology are considered; the coverage of the major demographic variables is less complete (age and secular time are hardly mentioned), and the chapters on applying our knowledge of risk factors in cancer control reflect the fact that, except in industry, this approach is still in its infancy, with many more possible than actual achievements to report. But to see what *is* possible, workers involved in the control of cancer as well as those studying its causation would do well to persue this book.

I. Leck